# Trend analysis of malaria prevalence in Halaba special district, Southern Ethiopia

**DOI:** 10.1186/s13104-019-4215-2

**Published:** 2019-03-29

**Authors:** Tsegaye Shamebo, Beyene Petros

**Affiliations:** Department of Microbial, Cellular and Molecular Biology (Infection Biology Stream), College of Natural and Computational Sciences, Addis Ababa University, Addis Ababa, Ethiopia

**Keywords:** Ethiopia, Halaba, Malaria prevalence, *Plasmodium* spp.

## Abstract

**Objective:**

The study aimed to determine the prevalence of malaria in Halaba special district, Southern Ethiopia, from 2013 to 2017.

**Results:**

Of a total 583,668 malaria suspected cases examined during the study period, 55,252 (9.5%) were microscopically confirmed to be positive for malaria, at the rate of 27,712 (50.2%) females and 27,540 (49.8%) males (P = 0.95). The highest prevalence of 8454 (15.3%) malaria cases were observed in Halaba health center, followed by Halaba district hospital, at 7290 (13.2%), while the lowest cases, 1765 (3.2%), were confirmed in Wejago health center. The highest prevalence of malaria, 25,716 (46.5%), was registered among the age group ≥ 15 year old (P = 0.006). *Plasmodium vivax* and *Plasmodium falciparum* were the two major malaria parasites detected in this study, with the prevalence of 33,855 (62.3%) and 21,397 (38.7%), respectively (P = 0.0001). The detected high prevalence of *P. vivax* in this study may clearly indicate that more attention has been given to control *P. falciparum* strains in the study area. This may be a great challenge for the achievement of malaria elimination goals. Therefore, all concerned bodies should act collaboratively to combat the high prevalence of *P. vivax* from the study district.

## Introduction

Malaria is major public health problems worldwide with an estimated 3.3 billion people is at risk of being infected with malaria and develop the disease and 1.2 billion are at high risk [1, 2]. Its burden is more concerning in poorest, children and pregnant women. Globally, an estimated 198 million cases and 584,000 deaths occurred due to malaria in 2013 [3]. This figure is showing a decrease in malaria case incidence by 30% and mortality rates by 47% since 2000 [4]. However, yet malaria remains a major public health problem in the world with significant medical, economic and social implications especially in sub-Saharan Africa countries [5]. Malaria is a major public health concern in Ethiopia. About 75% of the country’s landmass is malarious and 60% of the population is at risk of developing the disease [6]. Its prevalence and transmission in Ethiopia relay on altitude and rainfall [7]. In Ethiopia, most malaria cases occur at altitude < 2000 m above sea levels [8]. Its prevention and control strategies such as the use of insecticide treated bed-nets (ITNs), prompt and effective treatment of clinical cases, intermittent preventive treatment for pregnant women and under-five children, indoor residual spraying(IRS) are now being widely adopted across Ethiopia, with increasing amounts of coverage achieved [9]. However, the coexistence of both *Plasmodium falciparum* and *Plasmodium vivax* malaria parasites create a challenge in prevention, control and elimination of the disease [10–15].

Halaba special district is the known malaria endemic area in Ethiopia [16]. Unlike the national prevalence, the proportion of *P. vivax* and *P. falciparum* malaria parasites was 70% and 30% in the study district. In addition, the existence of anti-malaria drug-resistant *P. falciparum* strains was also reported from the same study area [17]. Hence, despite few studies have been conducted in Halaba special district, as the knowledge level of the investigators, there has not been studied report in the last 5 years to show the trend of malaria prevalence. Therefore, this study was aimed to investigate the trend analysis of malaria prevalence in Halaba special district, Southern Ethiopia, from 2013 to 2017.

## Main text

### Materials and methods

#### Study setting and period

**Fig. 1:**
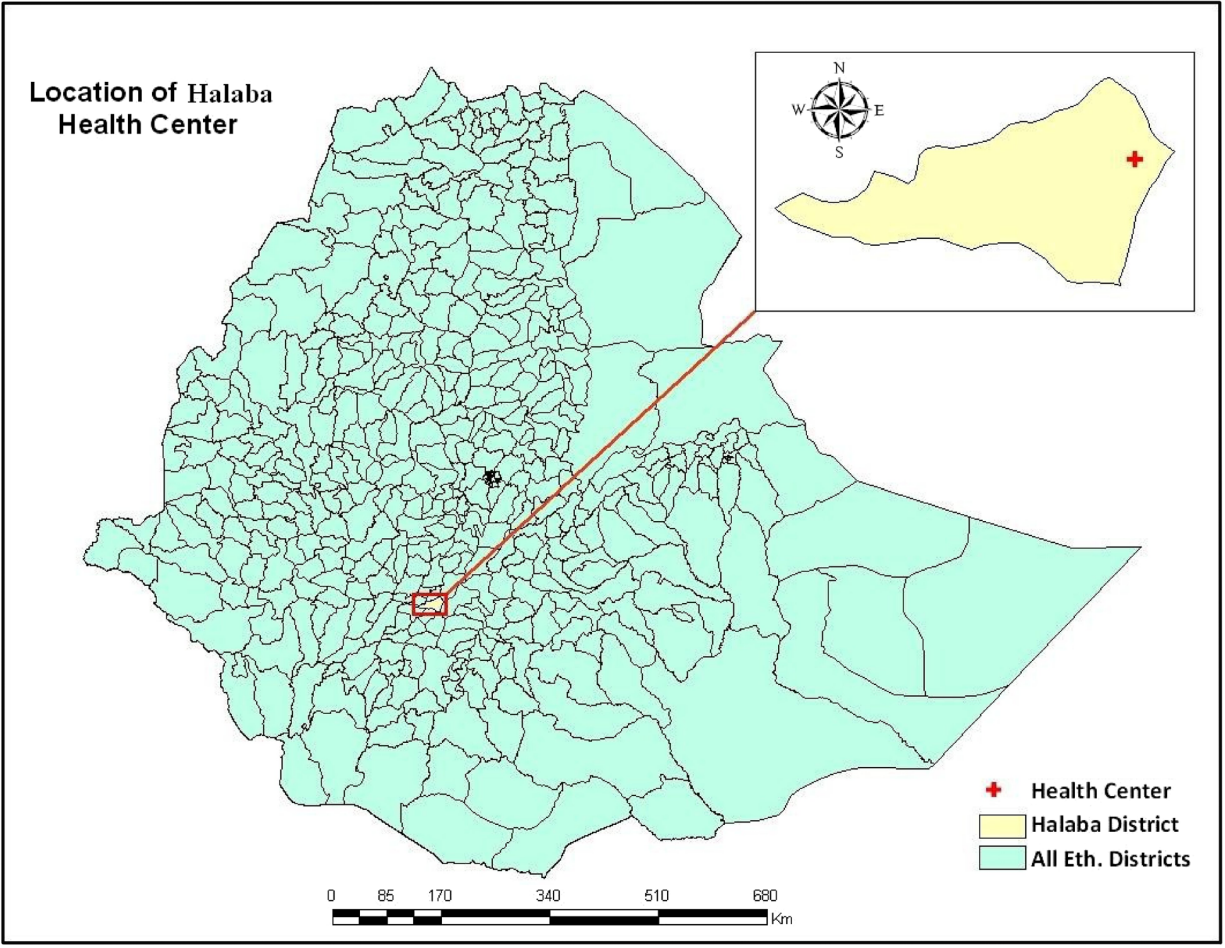
Map of the study area [8]

This study was conducted in Halaba special district, Southern Ethiopia. Halaba special district is located in Southern Nation, Nationalities and Peoples Region (SNNPR), at the distance of 85 km from Hawassa town and 310 km from Addis Ababa, the capital city of Ethiopia (Fig. 1). The study site is found within an altitude ranging from 1554 to 2149 m above sea level, and an astronomic location of 38°7′0″E longitude and 7°18′0″N latitude. Halaba special district is generally characterized by dry climatic condition with about 86% mid-land (Weinadega) and 14% law-land (Kola) zones. The mean annual rainfall of the study area is ranging from 857 to 1085 mm, while the mean annual temperature varies from 17 to 20 °C with a mean value of 18 °C. The district has a total population of 259,810, which encompasses 129,893 males and 129,917 females [8]. The study was conducted between March to May 2018.

#### Study design

Retrospective blood film malaria reports from Halaba special district hospital and health centers between September 2013 to August 2017 were carefully analyzed.

#### The study population and data collection

The study population was all malaria blood film tested individuals at Halaba special district hospital and health centers during the study period. To assess the participants’ condition, socio-demographic and laboratory data were collected from patients’ registration book. The meteorological data were collected from the nearby meteorological agency.

#### Data analysis

The data was entered into excel Microsoft and analyzed by using statistical package for social sciences software SPSS version 20.0. Pearson correlation analysis was used to evaluate the association between dependent and independent variables. In all cases *P* value < 0.05 was considered as statistically significant.

#### Ethical clearance

This study was conducted after obtaining the ethical clearance from the College of Natural and Computational Sciences, Addis Ababa University. Permission letter was also obtained from Halaba special district Health office to use the described retrospective malaria morbidity data.

### Results

**Fig. 2:**
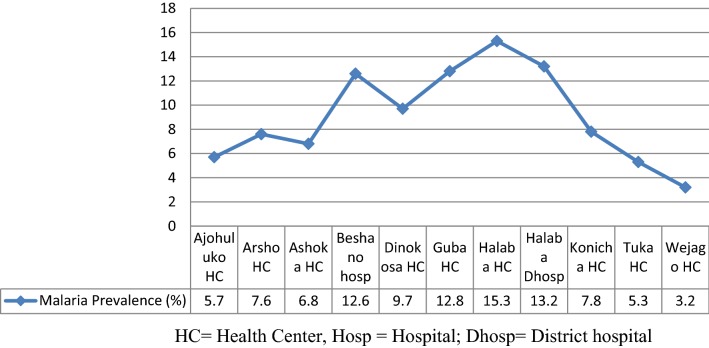
Distribution of malaria by different area in Halaba special district, Southern Ethiopia, 2013–2017

During the last 5 years period, from September 2013 to August 2017, a total of 583,668, blood smears were prepared and examined from malaria-suspected patients at Halaba special district health centers and hospitals for detection of the malaria parasite. Of these, 55,252 (9.5%) were microscopically confirmed malaria cases. Demographically, 27,540 (49.8%) of the patients were males and 27,712 (50.2%) were females. With regard to area, Halaba health center holed the highest (15.3%) malaria prevalence followed by Halaba district hospital (13.2%) and Guba health center (12.8%) while the lowest malaria cases were reported from Wejago health center (3.2%). There was a statistically significant variation (P = 0.002) in malaria prevalence among the areas (Fig. 2).

The highest malaria case was reported 41.2% in 2013 while the lowest case 5.1% was reported in 2017. However, the prevalence of *P. vivax* was seen increased, the lowest prevalence was 56.5% in 2013 and the highest prevalence was 67.2% in 2017, while the *P. falciparum* was observed decreased from 43.5% in 2013 to 5.1% in 2017. Overall, the prevalence of *P. falciparum* was 38.7% whereas that of *P. vivax* was 61.3% (Table 1).

**Table 1:** Distribution of *Plasmodium* species by year and meteorological data in Halaba special district, Southern Ethiopia, 2013–2017

Year	Total examined	Slide positive	*P. falciparum*	*P. vivax*	T-mean (c°)	R-mean (mm)	P value
2013	158,214	22,772 (41.2%)	9911 (43.5%)	12,861 (56.5%)	42.7	79.7	*0.0001*
2014	126,304	15,884 (28.8%)	6776 (42.7%)	9108 (57.3%)	42.9	62.7	*0.023*
2015	103,543	8785 (15.9%)	3551 (40.4%)	5234 (59.6%)	42.2	169.1	*0.004*
2016	86,006	4992 (9%)	1805 (36.2%)	3187 (63.8%)	47.3	70.4	*0.001*
2017	109,601	2819 (5.1%)	924 (32.8%)	1895 (67.2%)	50.6	185.3	*0.03*
Total	583,668	55,252 (9.5%)	21,397 (38.7%)	33,855 (61.3%)	45.1	113.4	–

Significant P values are given in italic (P < 0.05)

*T* Temperature,* R* Rainfall

With regard to meteorological data, at 1 month lag of rainfall, the prevalence of malaria was seen increased. There was a statistically significant variation (P = 0.0001) between malaria distribution and meteorological data (Table 1). There were no reported mixed infections during the study period. *P. vivax* was consistently more prevalent than *P. falciparum* irrespective of season.

Malaria was detected in all age groups in the study district but the age group ≥ 15 years were highly affected, with the prevalence rate of 25,716 (46.5%), followed by ≤ 4 year olds and 5–14 year olds with the prevalence rates of 17,231 (31.2%) and 12,305 (22.3%), respectively. There was statistical significant variation (P = 0.006) in malaria prevalence among the age groups. In all age groups *P. vivax* was seen predominant species. With regard to the sex, prevalence of 27,540 (49.8%) was reported for males and 27,712 (50.2%) for females. There was no statistical significant variation (P = 0.95) in malaria distribution among sex.

### Discussion

Malaria is a major public health concern in Ethiopia. About 75% of the country’s landmass is malarious and 60% of the population is at risk of developing the disease [6]. This retrospective study was aimed at evaluating 5-year trends of malaria prevalence in Halaba special district, Southern Ethiopia, from 2013 to 2017.

In the present study, the highest prevalence of malaria was treated in Halaba health center followed by Halaba district hospital and Guba health center whereas the lowest cases were confirmed in Wejago health center. The findings were higher than the previous report from the same study site by [18]. The climatic change and laboratory capacity to detect the parasite might be contributed to the existing variation.

The declining trend of malaria prevalence observed in the study district onwards 2013 was attributed to the existing better malaria prevention and control strategies [9]. The same trend has also been reported from different parts of the world including Eritrea [19], Zanzibar [20], Kenya [21] and South Africa [22] where proper usage of insecticide treated bed nets (ITNs) and indoor residual spraying (IRS) have reduced malaria prevalence. In agreement with the previous study [23] reported from Halaba, the prevalence of malaria, in the current study was slightly higher among females than males. In contrast, [6, 24] reported a higher malaria infection rate among males in northwest Ethiopia. The variation was may be due to a demographically large number of females in the current study area.

In the present study, malaria was detected in all age groups although the age group ≥ 15 years were highly affected, with the prevalence rate of 51,432 (46.5%), followed by ≤ 4-year-olds and 5–14-year-olds with the prevalence rates of 34,462 (31.2%) and 25,610 (22.3%), respectively. The finding was in agreement to what was recorded in Butajira area [25] and Halaba district [22] where a high prevalence of malaria in the age group 0–4 years and ≥ 15 years reported, respectively. This could be due to low immune status and less self-protection from vector among children and frequent outdoor spending of adults.

In the current study, the highest peak of malaria prevalence was registered in the months between (Sept–Dec) with the lowest reported prevalence in Jan, Feb, Jun, and Jul. This finding is in agreement with the studies reported from Ethiopia [10, 26], from China [27], and from India [28], where the positive correlation was observed between monthly rainfall and malaria parasite incidence. This may be because climatic and environmental factors other than rainfall could also determine the occurrence of malaria.

*Plasmodium falciparum* and *P. vivax* were the two species of malaria parasites detected in the current study. This was in line with the national profile of *Plasmodium* species [29]. However, the proportion of *P. falciparum* 38.7% and *P. vivax* 61.3% was quite different from the national prevalence of 60% and 40% for *P. falciparum* and *P. vivax*, respectively. In agreement with this, several studies [30] from Wonago [26] from Butajira [31] from South-central, Ethiopia [32] from Butajira [33] from China and [34] from India, reported a high prevalence of *P. vivax* in their studies. In contrast with this finding [6], from Gondar, [35] form Bahirdar [36] from Serbo town and [37] from Southwest Ethiopia reported a high prevalence of *P. falciparum*. This could be due to the difference in the study area, study period, climate, malaria control, and prevention strategies and laboratory capacities.

In this study, the overall malaria prevalence was dominated by *P. vivax*. The spread of chloroquine-resistant *P. vivax* may be one possible reason for the dominance of *P. vivax* in the study area [8, 38, 39]. The existence of chloroquine-resistant *P. vivax* has also been reported from the study district by different authors [40, 41]. Moreover, the high prevalence of *P. vivax* in the present study may also be an indication of the presence of people that are negative for Duffy antigen expression in Ethiopia ([42].

### Conclusion

Overall, the observed declining trend of malaria prevalence in Halaba special district onwards 2013 is suggestive for the existence of possible malaria control and prevention measures. But the existence of high prevalence *P. vivax* in the study district indicating that, much attention has been given to control *P. falciparum*. This could be a great challenge for the success of ongoing malaria elimination programme in Ethiopia. Therefore, concerned bodies should act aggressively in order to control the high prevalence of *P. vivax* in Halaba special district, Southern Ethiopia. Further detailed investigation emphasizing *P. vivax* chloroquine resistance pattern should also be needed.

## Limitation

Since this study was conducted using secondary data obtained from patients’ health record, being as any secondary data it is liable to disadvantages but we are familiar with the data set and critical analysis which the data was subjected to make the conclusion valid.

## Abbreviations

INRS: in-door residual spraying
ITNs: insecticide treated bed-nets
SNNPR: Southern Nation, Nationalities and Peoples Region
